# Histone H3K79 demethylation by KDM2B facilitates proper DNA replication through PCNA dissociation from chromatin

**DOI:** 10.1111/cpr.12920

**Published:** 2020-10-07

**Authors:** Joo‐Young Kang, Jin Woo Park, Ja Young Hahm, Hyeonsoo Jung, Sang‐Beom Seo

**Affiliations:** ^1^ Department of Life Science College of Natural Sciences Chung‐Ang University Seoul Korea

**Keywords:** cell cycle, chromatin, DNA replication, H3K79 demethylation, KDM2B, PCNA

## Abstract

**Objectives:**

The level of histone H3 lysine 79 methylation is regulated by the cell cycle and involved in cell proliferation. KDM2B is an H3K79 demethylase. Proliferating cell nuclear antigen (PCNA) is a component of the DNA replication machinery. This study aimed at elucidating a molecular link between H3K79me recognition of PCNA and cell cycle control.

**Materials and methods:**

We generated KDM2B‐depleted 293T cells and histone H3‐K79R mutant‐expressing 293T cells. Western blots were primarily utilized to examine the H3K79me level and its effect on subsequent PCNA dissociation from chromatin. We applied IP, peptide pull‐down, isothermal titration calorimetry (ITC) and ChIP experiments to show the PCNA binding towards methylated H3K79 and DNA replication origins. Flow cytometry, MTT, iPOND and DNA fibre assays were used to assess the necessity of KDM2B for DNA replication and cell proliferation.

**Results:**

We revealed that KDM2B‐mediated H3K79 demethylation regulated cell cycle progression. We found that PCNA bound chromatin in an H3K79me‐dependent manner during S phase. KDM2B was responsible for the timely dissociation of PCNA from chromatin, allowing to efficient DNA replication. Depletion of KDM2B aberrantly enriched chromatin with PCNA and caused slow dissociation of residual PCNA, leading to a negative effect on cell proliferation.

**Conclusions:**

We suggested a novel interaction between PCNA and H3K79me. Thus, our findings provide a new mechanism of KDM2B in regulation of DNA replication and cell proliferation.

## Graphical abstract

KDM2B prevents delay in dissociation of PCNA from chromatin during S phase via proper H3K79 demethylation. The timely destabilization of interaction between PCNA and replication origins caused by chromatin recruitment of KDM2B can contribute to replication fork progression and regulate DNA synthesis speed, leading to unperturbed cell cycle.

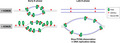

## 1 INTRODUCTION

Epigenetic modifications of histones regulate diverse cellular processes, such as transcription, DNA replication and DNA repair. Among those covalent modifications, histone H3 lysine 79 (H3K79) has been identified as a methylation target that is regulated by cell cycle, especially during S phase.^1^, ^2^, ^3^ The H3K79 methyltransferase DOT1L possesses enzymatic activity when nucleosomes, not free core histones, are used as substrates, implying its preference for chromatin substrates.^4^, ^5^ However, the molecular mechanism underlying the relationship between alteration of H3K79 methylation and S phase progression has not been elucidated.

Histone demethylase KDM2B is a JmjC domain‐containing protein, which has recently been identified as an H3K79 demethylase and is widely studied for its catalytic activity towards H3K36.^6^, ^7^, ^8^, ^9^ There have been several reports about KDM2B characteristics related to cell growth, cell proliferation, senescence and cell cycle progression.^6^, ^10^, ^11^, ^12^ Loss of KDM2B causes improper ESC differentiation, disruption of hematopoiesis, myeloid transformation, inhibition of cell proliferation, homeotic phenotype, developmental delay and embryonic lethality.^6^, ^13^, ^14^, ^15^, ^16^ Although the transcriptional regulation effects of KDM2B via H3K79 demethylation have been examined, it remains unknown whether other H3K79‐dependent biological effects could be under the control of KDM2B.

Proliferating cell nuclear antigen (PCNA) is an essential processivity factor for DNA replication by DNA polymerase. PCNA is also involved in the recruitment of many proteins associated not only with DNA replication but also with DNA repair.^17^, ^18^, ^19^ Intriguingly, a previous report revealed the interaction between PCNA and H3K56 methylation in the G1‐phase.^20^

Here, we show that H3K79 methylation in early S phase provides a platform for the recruitment of PCNA to sites where DNA synthesis is initiated. We further discovered the correlation between a gradual reduction in H3K79 methylation and PCNA dissociation from chromatin during S phase progression, which eventually ensures complete DNA replication. Our findings discerned a previously unknown mechanistic link between H3K79 methylation and cell cycle regulation based on the demethylase activity of KDM2B, which contributes to our understanding of the temporal control of DNA replication.

## 2 MATERIALS AND METHODS

### 2.1 Peptide pull‐down assay

Biotinylated trimethyl‐H3K79 peptides, H‐RLVREIAQDFK[me3]TDLRFQSSAVK[biotin]‐OH, dimethyl‐H3K79 peptides, H‐RLVREIAQDFK[me2]TDLRFQSSAVK[biotin]‐OH and unmodified‐H3K79 peptides, H‐RLVREIAQDFKTDLRFQSSAVK[biotin]‐OH, were purchased from AnaSpec (California, United States). Three μg peptides were pre‐bound to streptavidin‐Sepharose beads (GE Healthcare) and incubated overnight at 4°C with nuclear extract from 293T cells in binding buffer (20 mmol/L HEPES, pH 7.9, 150 mmol/L KCl, 1 mmol/L DTT, 1 mmol/L PMSF, 10% glycerol, 0.1% NP‐40). The particles were washed three times with 1 mL of washing buffer (20 mmol/L HEPES, pH 7.9, 150 mmol/L KCl, 1 mmol/L DTT, 1 mmol/L PMSF, 0.1% NP‐40). Peptide‐binding proteins were analysed by Western blot.

### 2.2 DNA fibre assay

DNA fibre assays to analyse replication fork progression were performed as described previously^21^ with some modification. Cells were labelled with 50 μmol/L 5‐iodo‐2’‐deoxyuridine (IdU, Glentham Life Science) for 40 minutes. 500 cells were placed on a microscope slide (Marienfeld Superior) in SB buffer (200 mmol/L Tris–HCl, pH 7.4, 50 mmol/L EDTA, 0.5% SDS). The slide was tilted to spread the DNA, and the DNA was fixed by methanol:acetic acid (3:1) mixture, followed by denaturation using 2.5 N HCl. The slide was incubated with anti‐BrdU antibody recognizing IdU to visualize IdU incorporation. Anti‐mouse Cy3 was used for secondary antibody. Images were captured with a Zeiss LSM 700 confocal microscope. Fibre length was measured using ImageJ software.

### 2.3 Isolation of proteins on nascent DNA (iPOND)

iPOND experiment was performed as described previously^22^ with some modification. Cells were treated with 2 mmol/L thymidine for 19 hours and released into S phase until 5 hours time point, and 5‐ethynyl‐2'‐deoxyuridine (EdU, Sigma) was added at 10 μmol/L for 20 minutes to yield a pulse‐labelled DNA. Cells were washed in PBS, crosslinked 20 minutes with 1% formaldehyde in PBS at RT, quenched for 20 minutes with 0.125 mol/L glycine in PBS, washed three times in cold PBS and harvested by scraping. Pellets were resuspended in permeabilization buffer (20 mmol/L HEPES, pH 7.5, 50 mmol/L NaCl, 3 mmol/L MgCl_2_, 300 mmol/L sucrose, 0.5% NP‐40), incubated on ice for 15 minutes and spun at 2500 *g* for 10 minutes. These pellets were washed in PBS, resuspended in Click Reaction buffer (10 μmol/L biotin‐azide, 10 μmol/L ascorbate, 2 mmol/L CuSO_4_ in PBS), incubated on ice for 1 h and washed in PBS. Pellets were resuspended in 400 μL Nuclear Lysis buffer (50 mmol/L Tris‐HCl, pH 8.0, 25 mmol/L NaCl, 2 mmol/L EDTA, 1% NP‐40), incubated on ice for 15 minutes, sonicated for total of 120 seconds and spun in a microcentrifuge at maximum speed for 10 minutes. Lysate was diluted with Wash buffer (50 mmol/L Tris‐HCl, pH 8.0, 150 mmol/L NaCl, 2 mmol/L EDTA, 0.5% NP‐40) and subjected to pull‐down with 30 μL streptavidin beads (GE Healthcare) overnight at 4°C. Beads were washed three times with Wash buffer. Nascent chromatin‐binding proteins were analysed by Western blot.

### 2.4 Statistical analysis

Data are expressed as mean ± SD of three or more independent experiments. Statistical significance (*P < .05, **P < .01, ***P < .001) was calculated using Microsoft Excel. Statistical evaluations were performed using Student's *t* test.

## 3 RESULTS

### 3.1 KDM2B‐mediated H3K79 demethylation regulates cell cycle progression

**FIGURE 1:**
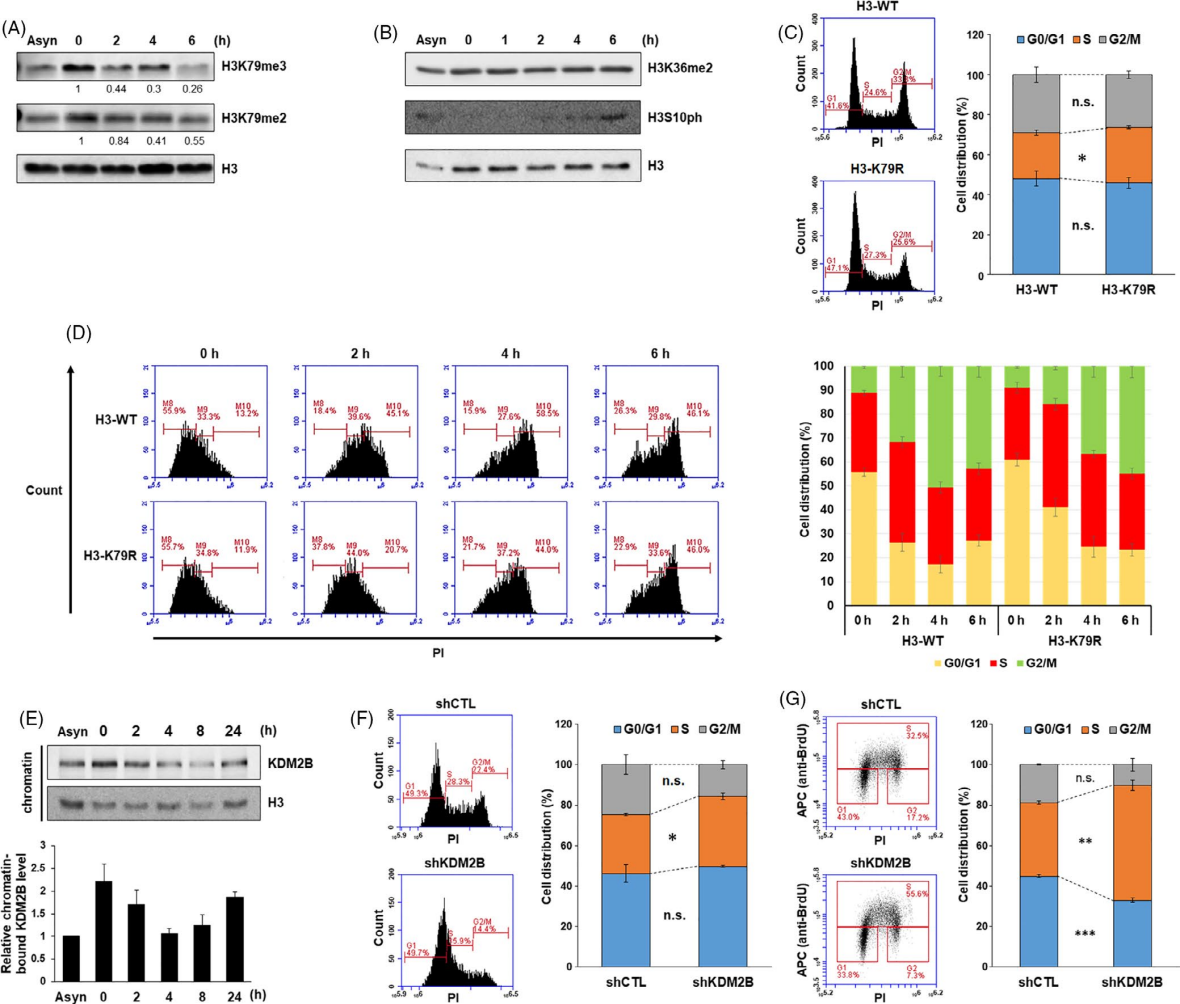
H3K79 demethylation caused by chromatin enrichment of KDM2B prevents S phase delay. A, 293T cells were arrested at the G1/S boundary by thymidine and then released into S phase. Quantification of H3K79 methylation level normalized to total H3 was based on the Western blot results. n = 1. B, 293T cells were arrested at the G1/S boundary by thymidine and then released into S phase. The levels of H3K36 methylation and H3S10 phosphorylation were analysed by Western blot of 3 independent experiments. C, 293T cells stably expressing H3‐WT or H3‐K79R were fixed and stained with propidium iodide (PI), and the DNA content was measured by fluorescence‐activated cell sorting (FACS). Data are presented as mean ± SEM from at least 5 experiments (*P < .05). D, 293T cells stably expressing H3‐WT or H3‐K79R were arrested at the G1/S boundary by thymidine and then released into S phase. Cells harvested at 0, 2, 4 and 6 h time points were fixed and stained with PI, and the DNA content was measured by FACS. Quantification of the cell distribution (mean ± SD) from 2 independent experiments. E, 293T cells were arrested at the G1/S boundary by thymidine and then released into S phase. The chromatin fractions were extracted, and the levels of KDM2B and histone H3 were measured by Western blot. Chromatin‐associated KDM2B levels were quantified using normalization to the loading control H3 from at least 3 independent experiments. F, shCTL and shKDM2B cells were fixed and stained with PI, and the DNA content was measured by FACS. Data are presented as mean ± SEM from at least 3 experiments (*P < .05). G, shCTL and shKDM2B cells treated with 10 μmol/L BrdU using pulse labelling for 15 min were fixed, immunostained with anti‐BrdU‐APC for 1 h and stained with PI. BrdU‐positive cells were measured by FACS. Data are presented as mean ± SEM from at least 3 experiments (**P < .01, ***P < .001)

Although regulation of H3K79 methylation in diverse model systems has been widely explored, detailed molecular insight into its function in cell cycle control in human cells has not yet been fully elucidated. To investigate H3K79 methylation dynamics during cell cycle progression, we examined changes in H3K79me2/3 following thymidine‐induced G1/S arrest and release into S phase. We observed that H3K79 methylation increased until S phase initiation and then decreased over time (Figure 1A, Figure S1A). Phosphorylation of H3S10 during G2/M was detected 6 hours after release into S phase, indicating the duration of S phase (Figure 1B). Next, we generated 293T cell lines stably expressing wild‐type H3 (H3‐WT) or the lysine‐to‐arginine mutation of H3 (H3‐K79R). Global H3K79 methylation levels declined by half after lentiviral infection with H3‐K79R (Figure S1C), leading to accumulation of cells in S phase (Figure 1C). To determine whether defective H3K79 methylation could disrupt proper S phase progression, we synchronized cells in early and late stages of S phase as well as in the G1/S boundary. We found that H3‐K79R delayed S phase completion compared to H3‐WT (Figure 1D). Given that expression of genes involved in cell cycle was not affected, we could rule out a transcriptional effect on cell cycle progression of H3‐K79R overexpression (Figure S1D). These data indicate that a cell cycle‐dependent fluctuation in H3K79 methylation is required for proper S phase progression.

To determine whether the H3K79 demethylase KDM2B is necessary for cell cycle progression, we performed chromatin fractionation and monitored the level of chromatin‐bound KDM2B during S phase. KDM2B was significantly enriched in chromatin immediately before S phase, remained bound to chromatin in early S phase, gradually dissociated from chromatin as cells pass through S phase and enriched again for the next round of cell cycle (Figure 1E); however, levels of KDM2B in the whole‐cell lysate unchanged (Figure S1E). This result indicates that KDM2B could be involved in DNA synthesis by affecting chromatin association of DNA replication factors, via H3K79 demethylation. To further characterize the role of KDM2B in cell cycle regulation, short hairpin RNAs, either a shCTL or shKDM2Bs, were introduced into cells to investigate the effect of KDM2B loss on S phase (Figure S1F). The results showed that a larger proportion of KDM2B‐deficient cells accumulated in S phase compared to control cells (Figure 1F). We also examined the ability of KDM2B‐deficient cells to perform DNA synthesis by analysing BrdU incorporation. Only about 36% of shCTL cells were BrdU‐positive, whereas more than 57% of shKDM2B cells were BrdU‐positive, implying the slowing of S phase and the impairment of DNA replication timing (Figure 1G). Interestingly, lack of KDM2B alone did not increase phosphorylated H2AX (γH2AX) levels, confirming that the retarded S phase observed in shKDM2B cells was not due to DNA damage checkpoint activation (Figure S1G). To rule out a transcriptional effect on cell cycle progression of KDM2B depletion, we analysed published RNA‐seq data of *Kdm2b*‐null mouse ES cells and found that KDM2B itself would not be associated with significant alteration in expression of cell cycle regulators (Figure S1H).^23^ These results indicate that KDM2B is enriched in the chromatin following initiation of DNA synthesis and regulates S phase progression through H3K79 demethylation.

### 3.2 PCNA interacts with H3K79 methylation

**FIGURE 2:**
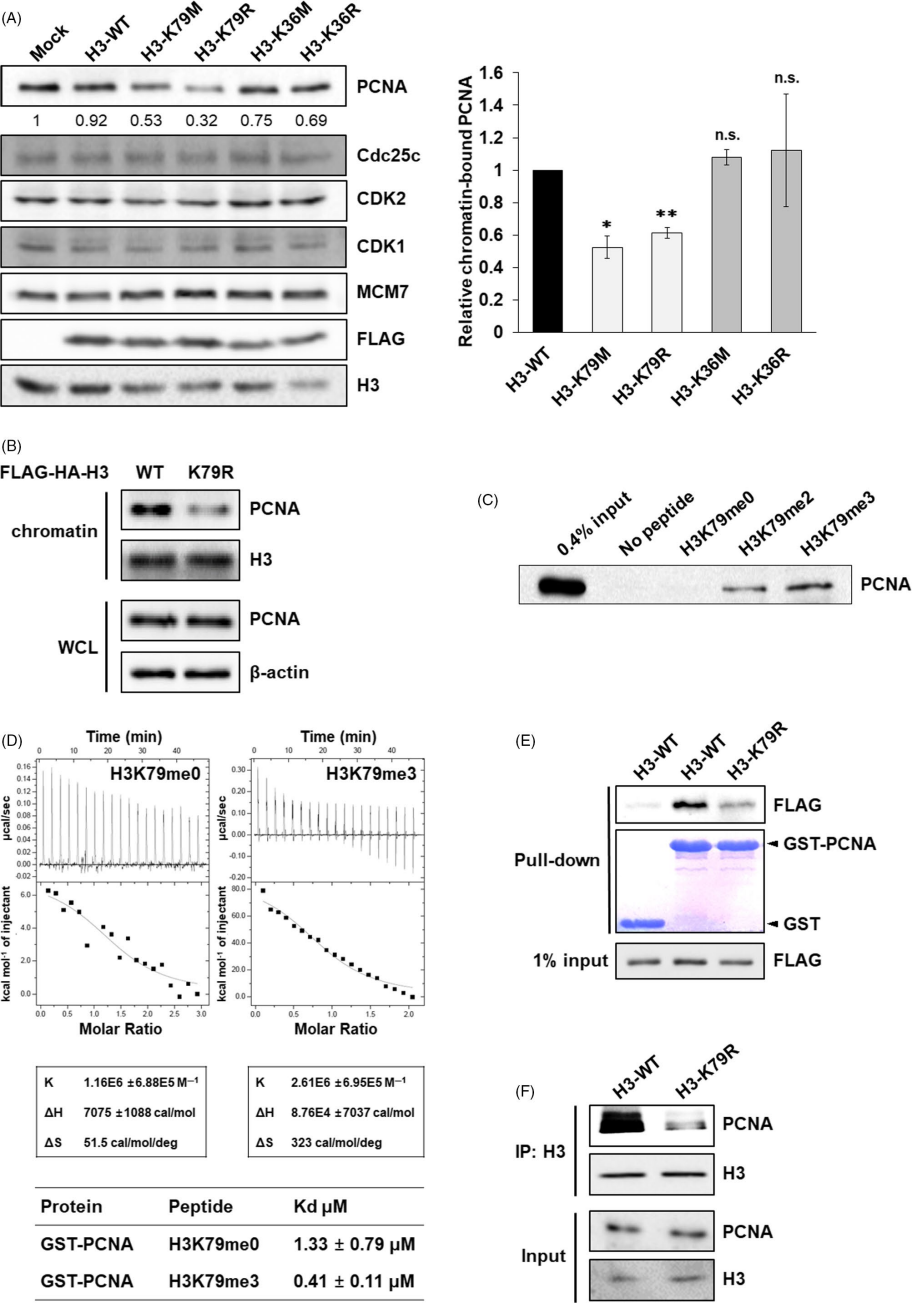
PCNA interacts with H3K79 methylation. A, Chromatin fractions were extracted from 293T cells transfected with FLAG‐tagged H3‐WT or different H3 mutants, and the levels of PCNA, Cdc25c, CDK2, CDK1 and MCM7 were measured by Western blot. Chromatin‐associated PCNA levels were quantified using normalization to the loading control H3. Data are presented as mean ± SEM from at least 3 experiments (*P < .05, **P < .01). B, Chromatin fractions were isolated from 293T cells stably expressing H3‐WT or H3‐K79R, and the indicated proteins were analysed by Western blot of 3 independent experiments. C, 293T cells were subjected to pull‐down using H3K79me0, H3K79me2 or H3K79me3 peptides (amino acids H3 69‐89) in whole‐cell lysate followed by Western blot. n = 1. D, Dissociation constants of GST‐PCNA with H3K79me3 or H3K79me0 peptides (amino acids H3 75‐83) were measured by isothermal titration calorimetry (ITC). E, Pull‐down of GST or GST‐PCNA in chromatin fractions isolated from 293T cells transfected with FLAG‐tagged H3‐WT or H3‐K79R. These results were repeated of at least 3 independent experiments. F, Immunoprecipitation of histone H3 and endogenous PCNA in 293T cells stably overexpressing H3‐WT or H3‐K79R. These results were repeated of at least 3 independent experiments

To explore the mechanism of delayed DNA replication by KDM2B depletion, we next analysed the chromatin binding of several DNA replication factors following transfection of FLAG‐tagged histone H3 containing K79 or K36 substitutions. We tested K36 mutants as a negative control to prove K79‐specific recruitment, since it has been previously shown that KDM2B also has demethylase activity towards H3K36.^24^ Of five proteins associated with the DNA replication machinery, PCNA levels in chromatin fractions of cells overexpressing K79 mutants were clearly decreased compared to those in WT cells or K36 mutants (Figure 2A), while chromatin incorporation levels of each FLAG‐H3 construct were not significantly different (Figure S2). Similarly, H3‐K79R cells showed considerably less chromatin‐bound PCNA compared to H3‐WT cells (Figure 2B). We also observed that H3K36 methylation unchanged during S phase, which rules out the possibility for H3K36‐mediated regulation of PCNA binding (Figure 1B, Figure S1B). To confirm the interaction between PCNA and methylated H3K79, we performed pull‐down assay using H3K79 peptide with whole‐cell lysate. PCNA strongly interacted with H3K79me3 peptide than H3K79me2 peptide, but not with unmethylated H3K79me0 peptide (Figure 2C). We also examined the binding affinity of purified GST‐PCNA to H3K79 peptides using isothermal titration calorimetry (ITC) analysis and confirmed it had a lower dissociation constant with H3K79me3 than with H3K79me0 (Figure 2D), suggesting that PCNA specifically binds to H3K79 in a methylation‐dependent manner. To investigate whether K79R mutation could destabilize H3K79‐PCNA interaction, we performed pull‐down assays using GST‐PCNA. The strong binding affinity between PCNA and H3 was dramatically reduced by the substitution of K79 (Figure 2E). Consistent with the data from in vitro protein‐binding analysis by pull‐down and ITC, immunoprecipitation assays revealed that the interaction between endogenous PCNA and H3 in the H3‐K79R‐incorporated chromatin fraction was greatly reduced compared to that of H3‐WT‐incorporated chromatin (Figure 2F), suggesting that H3K79 methylation is critical for PCNA association with chromatin and with histone H3 itself.

### 3.3 KDM2B regulates replication fork progression and PCNA dissociation via H3K79 demethylation

**FIGURE 3:**
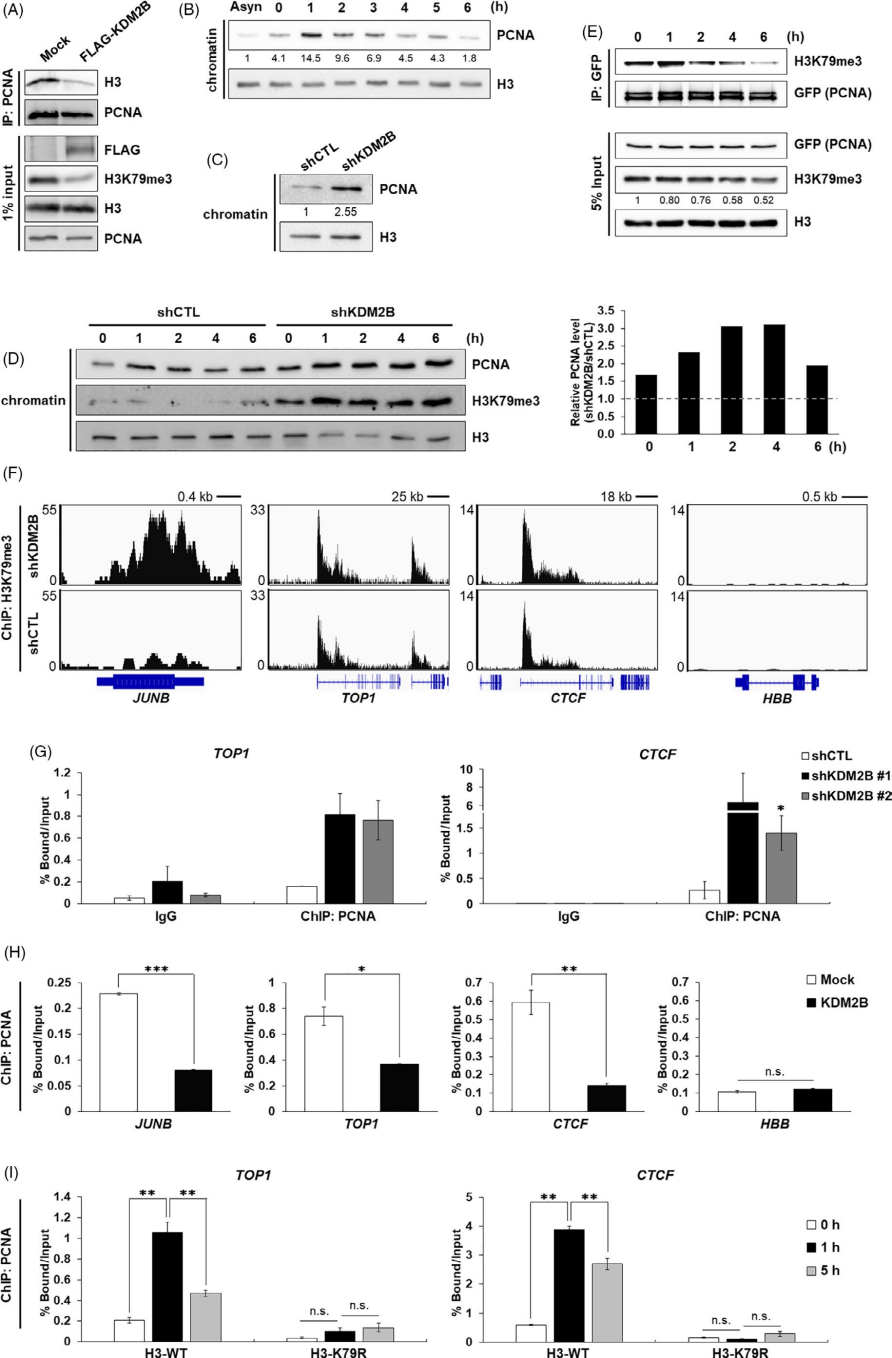
KDM2B depletion impairs PCNA dissociation and regulates replication fork progression. A, Whole‐cell lysates were prepared from 293T cells transfected with empty vector or FLAG‐KDM2B and immunoprecipitated with anti‐PCNA antibody. The disruption of interaction between PCNA and histone H3 was analysed by Western blot of 3 independent experiments. B, 293T cells were arrested at the G1/S boundary by thymidine and then released to S phase. The levels of PCNA and histone H3 in chromatin fractions were measured. Quantification of chromatin‐associated PCNA level normalized to chromatin‐associated H3 was based on the Western blot results. n = 1. C, shCTL or shKDM2B cells were arrested at G1/S and then released to S phase. After 1 h, cells were harvested and the levels of PCNA and histone H3 in chromatin fractions were measured. Quantification of chromatin‐associated PCNA level normalized to chromatin‐associated H3 was based on the Western blot results. n = 1. D, Chromatin fractions were isolated from shCTL or shKDM2B cells during S phase, and the levels of PCNA and H3K79me3 at indicated time points were analysed. Quantification of chromatin‐associated PCNA level normalized to chromatin‐associated H3 was based on the Western blot results and shown in a bar graph of a ratio of the values in shKDM2B and shCTL. n = 1. E, Immunoprecipitation of ectopically expressed GFP‐PCNA and endogenous H3K79me3 in whole‐cell lysates from synchronized 293T cells. Quantification of H3K79me3 level normalized to total H3 in input sample was based on the Western blot results. n = 1. F, H3K79me3 ChIP‐seq profiles over four regions of the genome in shCTL and shKDM2B cells. Below the sequencing traces, *JUNB*, *TOP1*, *CTCF* and *HBB* genes are indicated. G, Measurement of PCNA enrichment by ChIP‐qPCR analysis on two origins of replication. After KDM2B was depleted, *TOP1* and *CTCF* loci were immunoprecipitated and amplified using specific primers. Mean values ± SD from 3 independent experiments (*P < .05). H, Measurement of PCNA enrichment level by ChIP‐qPCR analysis on four origins of replication. After KDM2B was transfected into 293T cells, *JUNB*, *TOP1*, *CTCF* and *HBB* loci were immunoprecipitated and amplified using specific primers. Mean values ± SD from 3 independent experiments (*P < .05; **P < .01; ***P < .001). I, ChIP‐qPCR analysis for change in association and dissociation of PCNA on two origins of replication. After H3‐WT or H3‐K79R cells were arrested at G1/S and then released to S phase, *TOP1* and *CTCF* loci were immunoprecipitated and amplified using specific primers. Mean values ± SD from 3 independent experiments (**P < .01)

To determine whether KDM2B could affect the interaction between PCNA and H3, we transfected FLAG‐KDM2B and performed IP with chromatin fractions. Overexpression of KDM2B disturbed the binding of PCNA to H3 (Figure 3A). We next analysed the timing of PCNA association and found that recruitment of PCNA to chromatin peaked at 1 hour after S phase began. Following initiation of DNA synthesis, PCNA was gradually dissociated from chromatin (Figure 3B, Figure S3A). It was notable that the declining trends of both H3K79 methylation and chromatin‐bound PCNA during S phase were similar. We therefore tested chromatin localization of PCNA in KDM2B‐depleted cells. Surprisingly, shKDM2B cells had higher PCNA level in chromatin fraction than shCTL cells at early S phase (Figure 3C). To rule out PCNA‐loading activity of replication factor C (RFC) complexes, we analysed the largest subunit, RFC1. We observed that lack of KDM2B did not affect chromatin‐bound RFC1 level (Figure S4A**)**, suggesting that the retention of PCNA on the chromatin by KDM2B depletion is not dependent of clamp loader‐mediated process. Furthermore, immunocytochemistry revealed that KDM2B enrichment led to PCNA loss in chromatin, which was in contrast with PCNA accumulation in adjacent cells with low levels of KDM2B (Figure S4B); however, KDM2B depletion did not affect chromatin recruitment of RFC1 (Figure S4C). When we synchronized shCTL or shKDM2B cells at specific cell cycle phases and monitored changes in chromatin‐bound PCNA, loss of KDM2B increased H3K79me3 levels and caused retention of excessive PCNA throughout S phase (Figure 3D). To validate timely interaction between PCNA and methylated H3K79, we expressed GFP‐PCNA and confirmed that its binding to H3K79me3 was destabilized during S phase progression (Figure 3E). These data demonstrate that H3K79 demethylation by KDM2B is a mechanistic link underlying the temporal regulation of PCNA dissociation during cell cycle progression.

Our results showed that PCNA failed to become dissociated from chromatin in KDM2B‐depleted cells at the global level; therefore, we analysed the binding of PCNA at well‐studied replication origins using a chromatin immunoprecipitation (ChIP) assay. First, we compared H3K79me3 peaks in shCTL and shKDM2B cells from published ChIP‐seq data^8^ and selected four loci involved in DNA replication.^25^ The ChIP‐seq profiles revealed that KDM2B depletion upregulated H3K79me3 mark around the origins of replication, except for *HBB* locus (Figure 3F). We performed ChIP‐qPCR and demonstrated that PCNA was enriched at individual replication forks after KDM2B depletion (Figure 3G). To reveal that KDM2B‐mediated PCNA dissociation was not a result of transcriptional changes, we provided evidence that PCNA binding on replication origins was over 20 times stronger in S phase entrance, at which is not linked to high transcriptional activity (Figure S3B). Moreover, we analysed intergenic replication origins to interpret the relationship among DNA replication‐H3K79me transcription. Two intergenic replication initiation sites, intergenic region 1 (between *CRPCCP2* and *MST1P2*) and intergenic region 2 (between *PIK3CB* and *LINC01391*), were selected by combining nascent DNA sequencing data^26^ and H3K79me3 ChIP‐seq data^8^ (Figure S3C, D). Second, we expressed FLAG‐KDM2B and performed ChIP‐qPCR at these four loci during early S phase. Strikingly, KDM2B suppressed chromatin localization of PCNA at the loci of *JUNB, TOP1* and *CTCF*, due to reduced H3K79 methylation. In contrast, PCNA binding on the *HBB* locus, which was not decorated with H3K79me3, was not affected by KDM2B overexpression (Figure 3H). Third, we found that chromatin incorporation of H3‐K79R inhibited PCNA recruitment to replication origins, while PCNA promptly loaded onto these loci in H3‐WT cells following initiation of DNA synthesis (Figure 3I). To rule out possibility that association of PCNA would be derived from increased transcription, we performed ChIP assay using an antibody against RNA polymerase II and observed that both H3‐WT and H3‐K79R cells were transcriptionally repressive in early S phase (Figure S3E). H3K79 methylation levels at both intragenic and intergenic origins of replication reduced, validating the defective H3K79 methylation in H3‐K79R cells at replication origins (Figure S3F). Altogether, these data provide evidence that KDM2B facilitates PCNA dissociation from chromatin in replication initiation sites, by H3K79 demethylation.

### 3.4 Upregulated H3K79me level by KDM2B depletion has detrimental effect on DNA replication and cell proliferation

**FIGURE 4:**
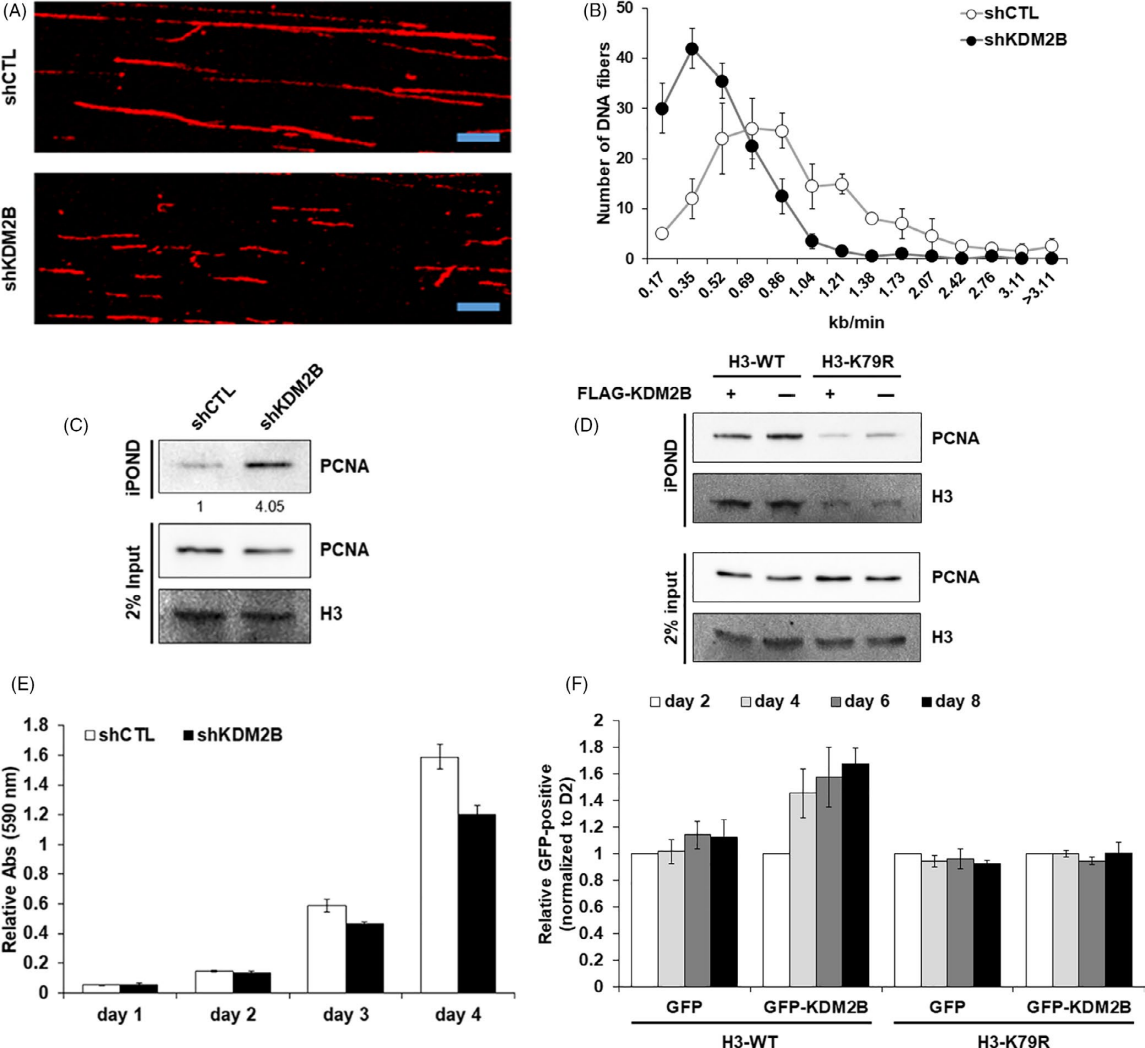
Depletion of KDM2B has detrimental effect on DNA replication and cell proliferation via H3K79 demethylation. A and B, Representative fluorescence images of DNA fibres isolated from shCTL or shKDM2B cells were shown (A), and distributions of the replication fork processivity rates were shown (B). Scale bar = 10 μm. Quantification of the fibre length (mean ± SD) from 2 independent experiments. At least 150 fibres were calculated. C, iPOND experiment comparing the PCNA levels from replication forks in control versus KDM2B‐depleted cells 5 h after thymidine release into S phase. Quantification of PCNA level associated with nascent DNA was based on the Western blot results. n = 1. D, iPOND experiment comparing the PCNA levels from replication forks in cells stably expressing H3‐WT or H3‐K79R with regard to KDM2B overexpression 5 h after thymidine release into S phase. The level of PCNA associated with nascent DNA was analysed by Western blot of 3 independent experiments. E, Cell viability of shCTL and shKDM2B cells was analysed by MTT assay of 2 independent experiments. F, GFP reporters were used in a lentiviral vector to indicate either empty or KDM2B lentiviral transduction. The percentage of GFP‐positive cells over time following transduction of empty lentiviral vector or KDM2B into stable cells overexpressing H3‐WT or H3‐K79R was analysed by FACS of 3 independent experiments. Cell proliferation was monitored for 8 d and normalized to the day 2 measurements

Based on the molecular mechanism by which loss of KDM2B promotes PCNA binding, we focused on DNA replication processivity and proliferative characteristics following KDM2B depletion. To this end, cells were pulse labelled with 5‐Iodo‐2'‐deoxyuridine (IdU), a thymidine analog, for DNA fibre analysis. We measured DNA fibre length and compared the replication rate of shCTL and shKDM2B cells. KDM2B‐depleted cells had larger numbers of short nascent DNA tracks compared to control cells (Figure 4A, B). To assess the replication forks‐specific PCNA retention, KDM2B‐deficient cells were analysed using isolation of proteins on nascent DNA (iPOND) experiment that permits a quantitative analysis of proteins on chromatin following DNA replication.^22^, ^27^ We treated cells with the nucleoside analog 5‐ethynyl‐2'‐deoxyuridine (EdU) by pulse labelling and found that PCNA on replicating chromatin was still not dissociated at 5 hours after release into S phase in shKDM2B cells, showing the temporal defect in newly synthesized DNA (Figure 4C). In addition, in late S phase, PCNA had little association with chromatin in H3‐K79R cells regardless of KDM2B overexpression, while ectopic expression of KDM2B promoted PCNA dissociation from nascent chromatin in H3‐WT cells (Figure 4D). These experiments indicated that KDM2B contributes to the maintenance of a proper chromatin landscape for processive DNA replication at an appropriate speed.

To demonstrate the impact on cell proliferation, we next assessed cell viability using MTT assay. KDM2B‐deficient cells displayed slower proliferation than control cells (Figure 4E). To verify whether the inhibitory effect of KDM2B knockdown is attributable to impaired H3K79 demethylation, we transduced lentiviral vector containing both a GFP reporter and a KDM2B construct into H3‐WT or H3‐K79R cells and sorted GFP‐positive cells using flow cytometry analysis. Remarkably, cells with overexpressed KDM2B showed enhanced cell proliferation for 8 days following viral infection relative to cells transduced with the empty vector, suggesting that KDM2B promotes cell proliferation. Moreover, this enhancement of cell proliferation by KDM2B was weakened in H3‐K79R cells (Figure 4F). These data indicate that KDM2B is required for unperturbed proliferation and cell cycle progression by ensuring timely DNA replication via H3K79 demethylation during S phase. Abrogation of proliferative control caused by aberrant hyper‐ or hypomethylation of H3K79 reflects this improper PCNA displacement due to H3K79‐dependent binding.

## 4 DISCUSSION

The decline in H3K79 methylation during S phase has been extensively studied, and the presence of demethylase has been suggested. Given that H3K79 demethylase activity of KDM2B has been characterized and that H3K79 methylation associates with origins of replication,^1^, ^8^ we wondered how KDM2B contributes to the molecular events that occur in the chromatin during DNA replication. Because the highest level of H3K79 methylation is a prerequisite for G1/S transition,^3^ we expected that H3K79 demethylation would be necessary for S/G2 transition.

While DNA is replicated, it is nucleosome‐free. Although the histone octamers are lost from DNA during DNA replication, the nucleosomes are immediately reassembled into chromatin using both parental and newly synthesized histones after replication fork passes.^28^ Histone proteins, which are marked with H3K9me, H3K56ac, H4K91ac and diacetylation of H4K5 and H4K12, respectively, affect the replication‐coupled nucleosome assembly.^29^ However, despite its critical roles in replication initiation and cell cycle regulation of DOT1L‐mediated H3K79 methylation,^1^, ^2^ a detailed mechanism by which the oscillation of H3K79 methylation levels by itself controls the smooth passage of replication forks and drives cell cycle progression has not been investigated yet. Although previous works have reported that transcribed regions were correlated with replication initiation sites and that H3K79me2 existed in the near replication initiation sites,^1^, ^26^, ^30^ we found intergenic regions which were decorated with H3K79 methylation and had a functional role as replication origins, based on a published sequencing data of nascent strands.^26^ These intergenic origins might suggest a universal role of PCNA‐H3K79me interaction in regulation of DNA replication and S phase progression, whereas genome‐wide analysis is still needed.

In considering aspects of DNA replication timing, H3K56me1 has been reported to form a pool of PCNA in G1‐phase to prime PCNA to bind at sites where DNA synthesis initiates.^20^ Since no histone mark that is responsible for PCNA anchoring in chromatin during S phase has been elucidated thus far, we aimed to discern whether fluctuating H3K79 methylation could be a determinant of PCNA dynamics related to chromatin association and dissociation. Previous studies have revealed that H3K79 residue is within the globular domain of histone H3.^4^, ^31^ Strikingly, since K56 is also located in the globular core of histone H3,^32^, ^33^ we speculated about the structural commonality between H3K79 and H3K56 residues. Moreover, the biological roles of H3K79me and H3K56me in DNA replication might support the potential structure‐function relationships existing between the two residues.^1^, ^3^, ^5^, ^20^ The docking mode by which PCNA switches its interacting histone mark from H3K56me to H3K79me after S phase entry appears reasonable, since H3K56me1 levels do not change during cell cycle progression, in contrast to H3K79 methylation level. Indeed, PCNA binds a wide range of chromatin‐associated factors and there are some examples for regulation of PCNA‐interacting proteins by post‐translational modifications (PTMs), including Pol η and FEN1.^34^, ^35^, ^36^ Our data show how two different PTMs, H3K56me in G1‐phase and H3K79me in S phase, could contribute to successful initiation‐progression‐completion of DNA replication.

Based on these findings, we envision the following mechanism to explain why passive H3K79 demethylation following KDM2B depletion leads to S phase delay and retardation of cell proliferation. Dysregulation of H3K79 methylation in pathological and tumorigenic pathways has been studied extensively.^3^, ^37^ Although aberrant H3K79 hypermethylation due to lack of KDM2B appears to be the opposite of the loss of H3K79 methylation concomitant with DOT1L deficiency, it is rational to consider these two contexts as similar from the aspect of perturbed H3K79 methylation. Thus, in terms of the regulators of PCNA localization at chromatin via H3K79 methylation status, knockdown of either DOT1L or KDM2B similarly shows negative effect on cell proliferation as well as on DNA replication.

**FIGURE 5:**
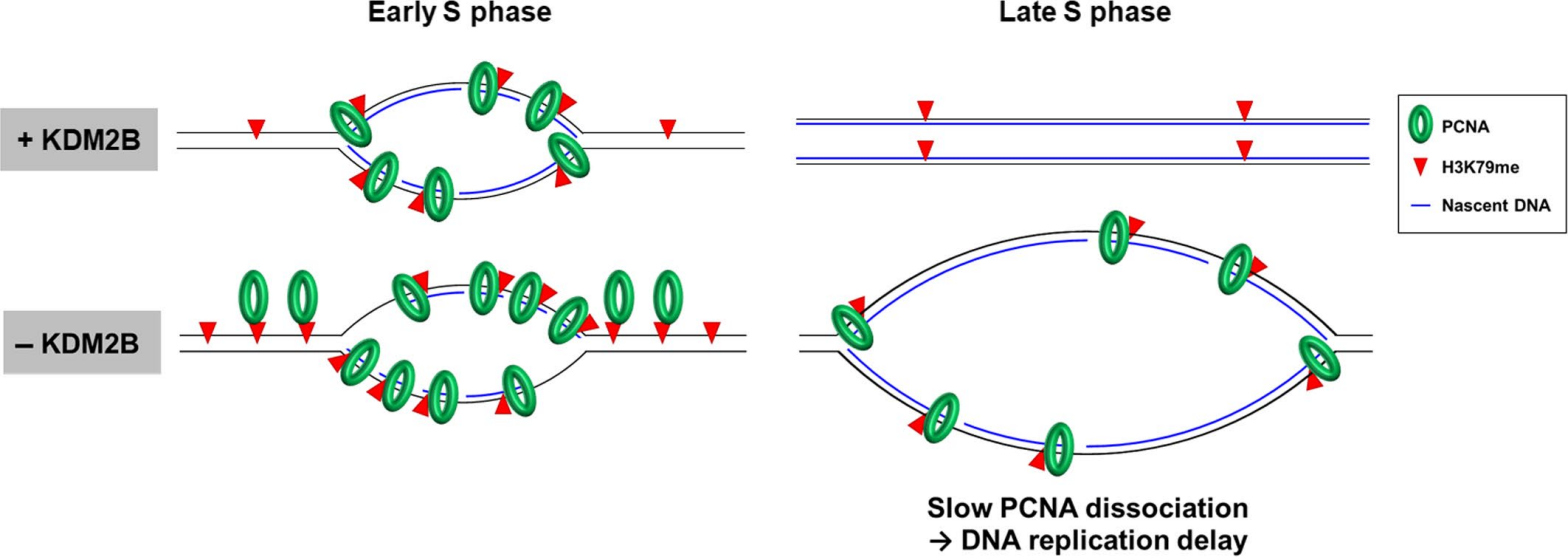
Schematic model shows that KDM2B prevents delay in dissociation of PCNA from chromatin during S phase via proper H3K79 demethylation, and this may contribute to timely cell cycle progression

We propose a model to explain the replication fork progression and DNA synthesis speed in KDM2B‐depleted condition (Figure 5). During S phase, recruitment of KDM2B to chromatin reduces H3K79 methylation that in turn leads to PCNA dissociation when DNA replication is completed. In conditions of KDM2B deficiency, excessive PCNA accumulates near the replication fork and it is slowly dissociated from chromatin due to a lack of timely H3K79 demethylation. Through our study, we discovered that cell cycle‐dependent changes in H3K79 methylation, together with the H3K79 demethylase KDM2B and the replication factor PCNA, play an essential role in S phase regulation. Identifying another histone mark as a docking site for PCNA and the histone‐binding motif within PCNA may give insight into unknown functions of epigenetic modifications in DNA replication.

## CONFLICTS OF INTEREST

The authors declare no conflict of interest.

## AUTHOR CONTRIBUTIONS

S.‐BS and J.‐YK designed the experiments and analysed the results. J.‐YK, JWP, JYH and HJ carried out the experiments. S.‐BS and J.‐YK wrote the manuscript with input from all authors.

## Supporting information

FigS1‐S4

Supplementary material

## Data Availability

The authors declare that the data supporting the findings of this study are available from the corresponding author upon reasonable request.
