# Supplementary figures and images for: Histone H3K79 demethylation by KDM2B facilitates proper DNA replication through PCNA dissociation from chromatin
Source: Cell Prolif. 2020 Oct 7;53(11):e12920. doi: 10.1111/cpr.12920 (PMC7653264; doi:10.1111/cpr.12920)

FIGURE S1.


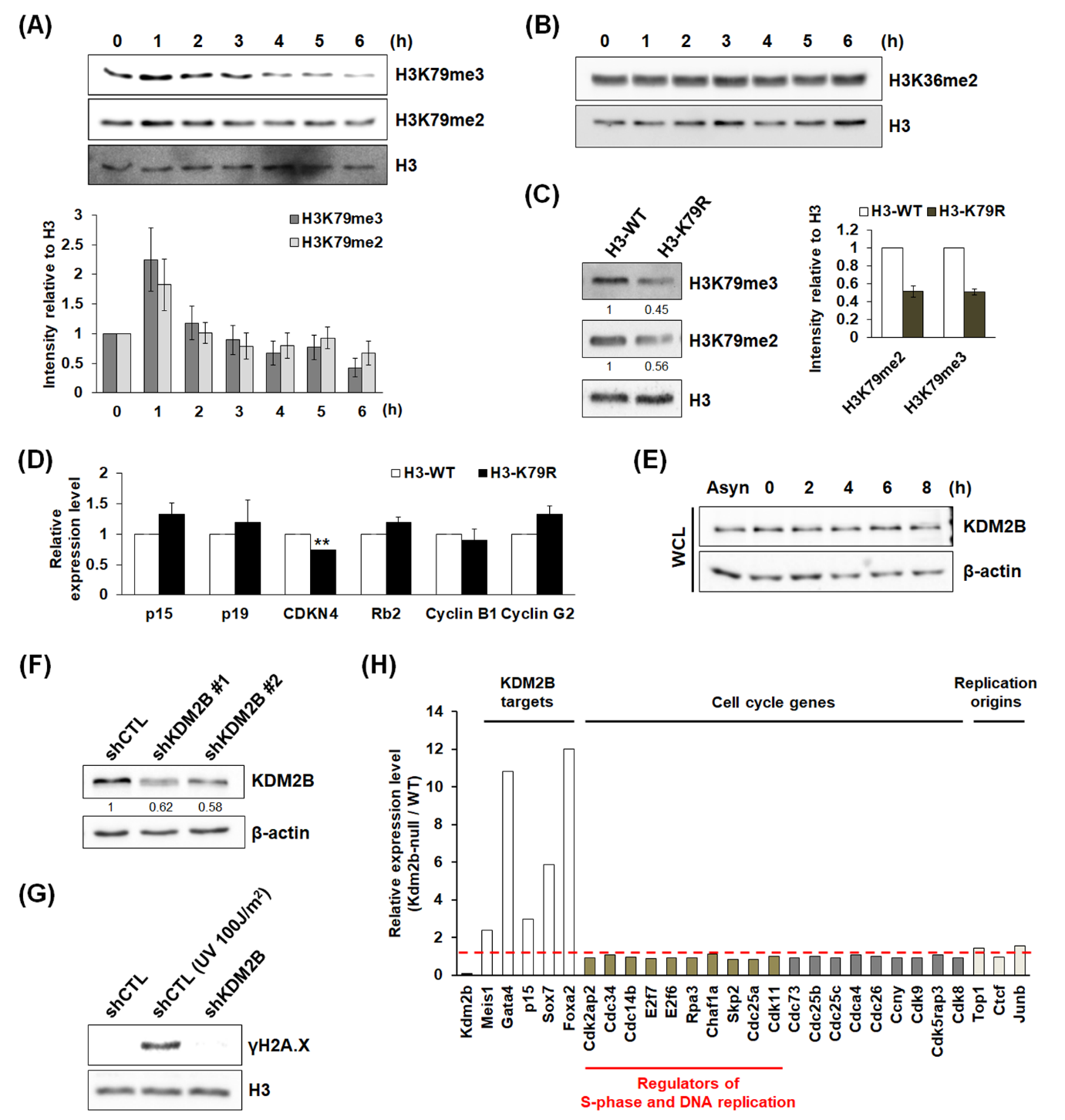


FIGURE S2.


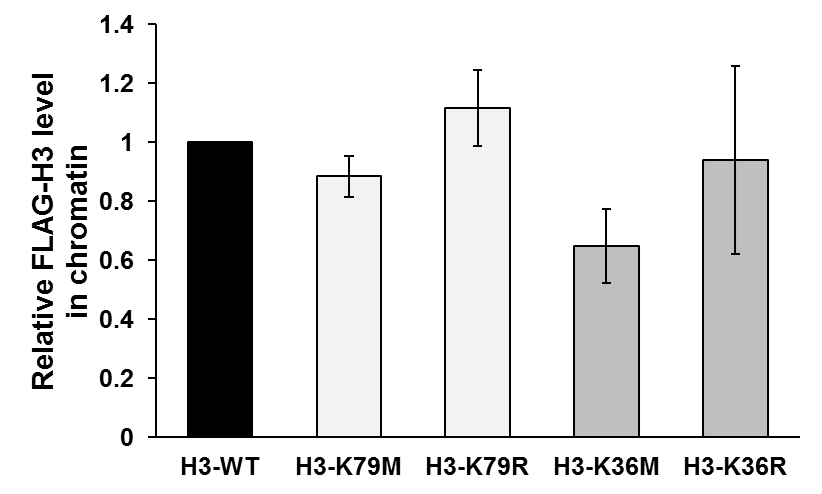


FIGURE S3.


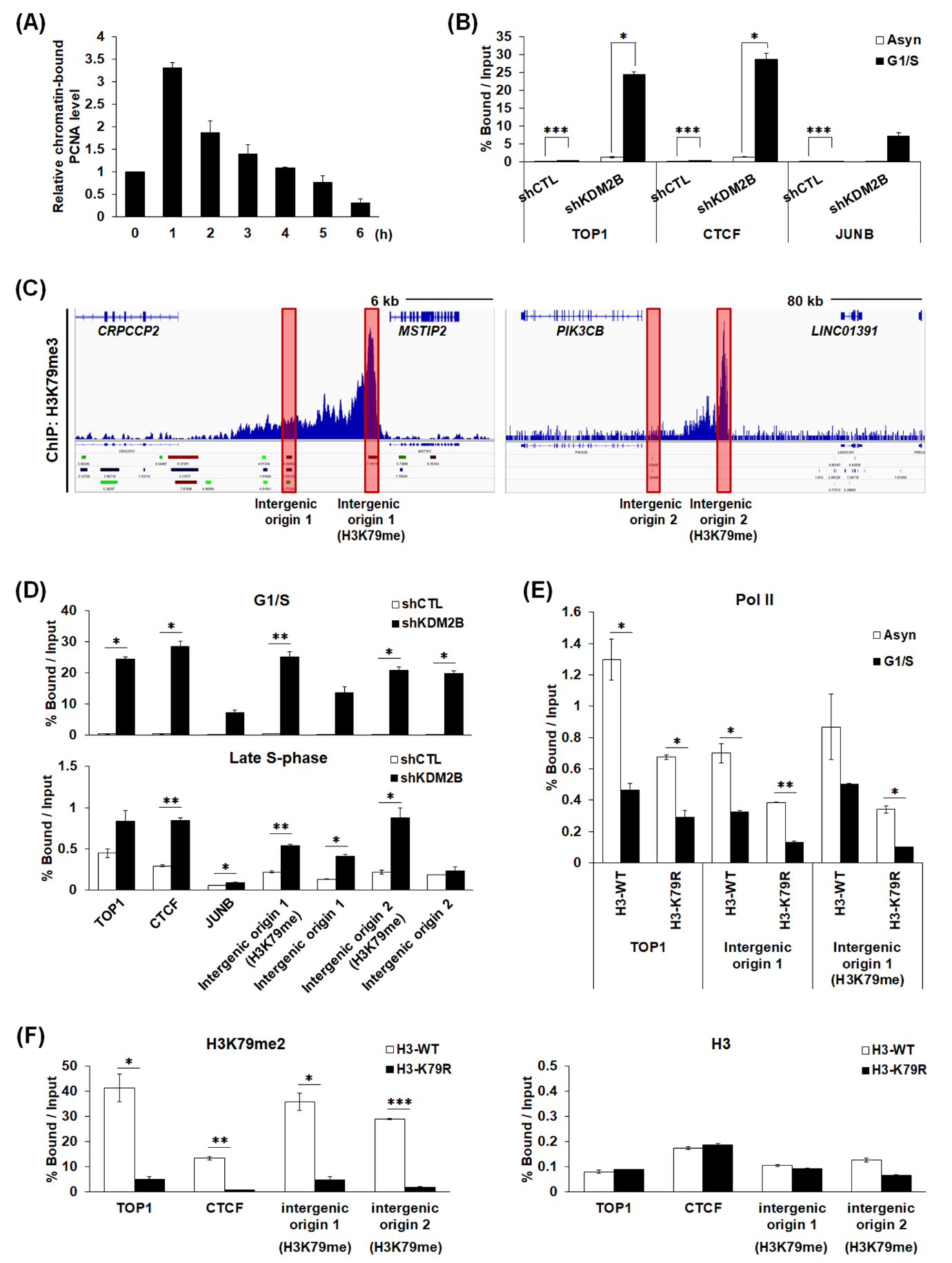


FIGURE S4.


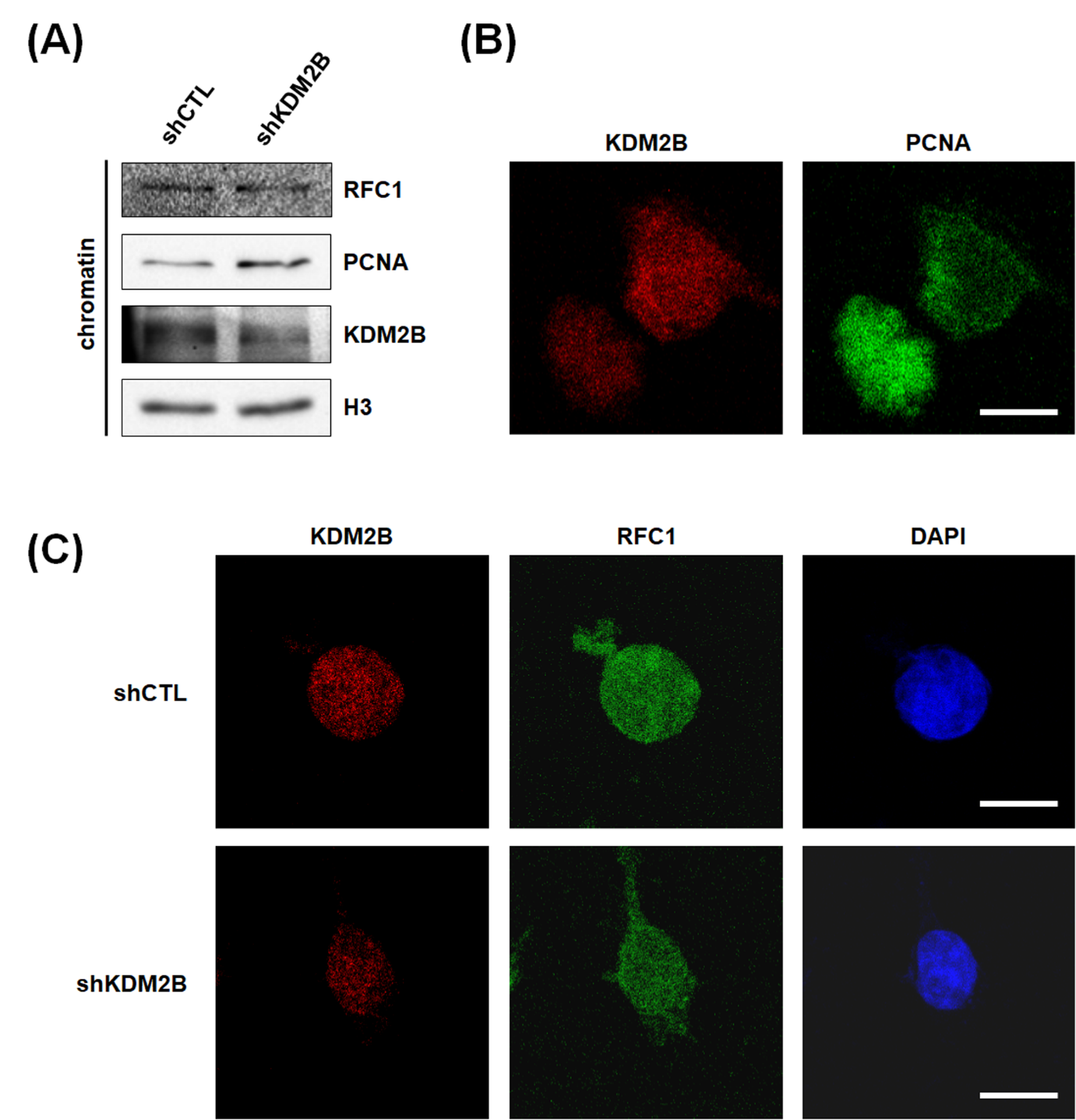

Supplement: Supplementary file 1 — FigS1‐S4 [file CPR-53-e12920-s001.docx]
