# Supplementary material for: Histone H3K79 demethylation by KDM2B facilitates proper DNA replication through PCNA dissociation from chromatin
Source: Cell Prolif. 2020 Oct 7;53(11):e12920. doi: 10.1111/cpr.12920 (PMC7653264; doi:10.1111/cpr.12920)
Supplement: Supplementary file 2 — Supplementary material [file CPR-53-e12920-s002.docx]

**Supplementary Information**

**Histone H3K79 demethylation by KDM2B facilitates proper DNA replication through PCNA dissociation from chromatin**

Joo-Young Kang,^1^ Jin Woo Park,^1^ Ja Young Hahm,^1^ Hyeonsoo Jung,^1^ Sang-Beom Seo^1,*^

**Supplementary Information:**

Supplementary Figure Legends for Figures S1-S4;

Supplementary Table S1;

Supplementary Procedures.

**SUPPLEMENTARY FIGURE LEGENDS**

**FIGURE S1.** A, 293T cells were arrested at the G1/S boundary by thymidine and then released into S phase. The immunoblots from three independent experiments were quantified and displayed in a bar graph of the level of H3K79 methylation normalized to total H3. B, 293T cells were arrested at the G1/S boundary by thymidine and then released into S phase. The level of H3K36 methylation is indicated. C, Chromatin fractions were isolated from 293T cells that stably express H3-WT or H3-K79R mutant and subjected to histone analysis by Western blot with the indicated antibodies. The immunoblots from three independent experiments were quantified and displayed in a bar graph of the level of H3K79 methylation normalized to total H3. D, RT-qPCR analysis of cell cycle-related gene expressions normalized using *GAPDH* in cells that stably express H3-WT or H3-K79R mutant. All error bars indicate SEM for at least triplicate experiments. ***P* < 0.01. E, 293T cells were arrested at the G1/S boundary by thymidine and then released into S phase. The whole-cell lysates were extracted, and the level of KDM2B was measured by Western blot of three independent experiments. F, 293T cells were infected with lentivirus expressing shCTL or shKDM2B. After 3 days of selection in puromycin, KDM2B level was measured. Quantification of KDM2B level normalized to β-actin was based on the Western blot results. *n* = 1. G, The level of γH2AX in shCTL and shKDM2B cells was analyzed by Western blot. Cells treated with 100 J/m^2^ UV showed induction of DNA damage checkpoint activation. H, RNA-seq data of *Kdm2b*-null mouse ES cells (Boulard, M. *et al*., 2015) showing the expression of genes related to cell cycle control and DNA replication regulation.

**FIGURE S2.** Chromatin fractions were extracted from 293T cells transfected with FLAG-tagged H3-WT or different H3 mutants, and the level of FLAG-H3 were measured by Western blot. The immunoblots from three independent experiments were quantified and displayed in a bar graph of the chromatin-incorporated FLAG-H3 level normalized to H3 intensity.

**FIGURE S3.** A, 293T cells were arrested at the G1/S boundary by thymidine and then released into S phase. The chromatin-bound PCNA level measured by three independent Western blots were quantified and displayed in a bar graph following normalization to H3. B, Measurement of PCNA enrichment by ChIP-qPCR analysis on three replication origins. After shCTL and shKDM2B cells were arrested at G1/S border, *TOP1*, *CTCF*, and *JUNB* loci were immunoprecipitated and amplified using specific primers. Mean values ± SD from 2 independent experiments. **P* < 0.05; ****P* < 0.001. C, H3K79me3 ChIP-seq profiles (upper) combined with a nascent DNA sequencing data of human cell lines (lower) (Martin, M, M. *et al*., 2011) showing two intergenic replication initiation sites across the genome. Above the sequencing traces, *CRPCCP2*, *MST1P2*, *PIK3CB*, and *LINC01321* loci are indicated. Four primer regions used in ChIP-qPCR analysis are represented in red boxes. D, ChIP-qPCR analysis for accumulation of PCNA on both intragenic and intergenic origins of replication. After shCTL and shKDM2B cells were arrested at G1/S and then released into S phase, seven loci were immunoprecipitated and amplified using specific primers. Mean values ± SD from 2 independent experiments. **P* < 0.05; ***P* < 0.01. E, Measurement of Pol II recruitment by ChIP-qPCR analysis for reduction in transcription on replication initiation sites. After H3-WT and H3-K79R cells were arrested at G1/S border, three loci were immunoprecipitated and amplified using specific primers. Mean values ± SD from 2 independent experiments. **P* < 0.05; ***P* < 0.01. F, ChIP-qPCR analysis for H3K79me3 and histone H3. Four loci of replication origins in H3-WT cells or H3-K79R cells were analyzed. Mean values ± SD from 2 independent experiments. **P* < 0.05; ***P* < 0.01; ****P* < 0.001.

**FIGURE S4.** A Chromatin fractions were extracted from shCTL and shKDM2B 293T cells, and the levels of RFC1, PCNA, and KDM2B were measured by Western blot. B, Immunocytochemistry of 293T cells. Changes in signal intensity were detected after double immunostaining with anti-KDM2B and anti-PCNA antibodies. Scale bar = 10 μm. C, Immunocytochemistry of shCTL and shKDM2B 293T cells. Changes in signal intensity were detected after double immunostaining with anti-KDM2B and anti-RFC1 antibodies. Scale bar = 10 μm.

**Supplementary Table S1.** Regions examined by PCNA, Pol II, H3K79me2, and H3 ChIP.

| Primer Set | Chromosomal Location | Primer Set Description |
| --- | --- | --- |
| 1 | Forward: ATAAAGGCGTGTGGCTCAGG  Reverse: ATAGCTTTCCTGGCGTCGTT | *JUNB* locus |
| 2 | Forward: CAGTGAGCCCAAATGCGAAC  Reverse: GAGACTCCAGAAACGGCTGA | *TOP1* locus |
| 3 | Forward: TTCCCTTATCAGCACCCGC  Reverse: GCACGGTTTAATCGCTCCAC | *CTCF* locus |
| 4 | Forward: CAACTCCTAAGCCAGTGCCAGAAG  Reverse: TGCCCTGACTTTTATGCCCAGC | *HBB* locus |
| 5 | Forward: ACCTTTCATGCCAGCTCCTACCC  Reverse: AGTGGCTTACACTGGGGGAATA | Intergenic replication origin 1 |
| 6 | Forward: ACCTTCCAGACACCCACTCCG  Reverse: ACGGGATAGCCGTAGCAGG | Intergenic replication origin 1 (H3K79me) |
| 7 | Forward: GGCATCCCCACACTCCTTAG  Reverse: AGACTGGGTGCCGACAATAG | Intergenic replication origin 2 |
| 8 | Forward: CCACCACCTTCGGAGTGTTT  Reverse: TGGCTGACATTCACAAAGCA | Intergenic replication origin 2 (H3K79me) |

**SUPPLEMENTARY PROCEDURES**

**Plasmid constructs**

p3XFLAG-CMV-KDM2B, pLenti-puro-H3-WT, pLenti-puro-H3-K79R, p3XFLAG-CMV-H3-WT, p3XFLAG-CMV-H3-K79M, p3XFLAG-CMV-H3-K79R, p3XFLAG-CMV-H3-K36M, p3XFLAG-CMV-H3-K36R, pIRES-FLAG-HA-H3-WT, pIRES-FLAG-HA-H3-K79R, and pEGFP-PCNA were used in each experiment.

**Cell culture**

293T cells were grown in DMEM that contained 10% heat-inactivated fetal bovine serumand 0.05% penicillin-streptomycin at 37°C in a 5% CO_2_ atmosphere. For cell synchronization, thymidine (2 mM; Sigma) was treated for 19 h.

**Antibodies**

Antibodies used in this study were as follows: anti-KDM2B (09-864; Millipore), anti-beta-actin (sc-47778; Santa Cruz Biotechnology), anti-FLAG(F3165; Sigma-Aldrich), anti-histone H3 (05-499; Millipore), anti-H3K79me3 (ab2621; Abcam), anti-H3K79me2 (ab3594; Abcam), anti-H3K36me2 (ab9049; Abcam), anti-H3S10ph (9701; Cell Signaling Technology), anti-gamma H2A.X (phospho S139) (ab2893; Abcam), anti-PCNA (sc-56; Santa Cruz Biotechnology), anti-Cdc25c (sc-13138; Santa Cruz Biotechnology), anti-CDK2 (sc-6248; Santa Cruz Biotechnology), anti-CDK1 (sc-54; Santa Cruz Biotechnology), anti-MCM7 (sc-9966; Santa Cruz Biotechnology), anti-RFC1 (sc-390948; Santa Cruz Biotechnology), anti-GFP (sc-9996; Santa Cruz Biotechnology), and anti-BrdU (347580; BD Bioscience).

**Stable knockdown cell lines**

DNA oligonucleotides that encode KDM2B short hairpin RNA (shRNA) #1 (5’-CCTGAGGAAGAAGCGGAAATA-3’) and KDM2B shRNA #2 (5’-GACTGCAATAAGGTCACTGAT-3’) were subcloned into the pLKO.1-puro lentiviral vector (Addgene, Cambridge, MA, USA), according to standard procedures. To produce virus particles, 293T cells were cotransfected with plasmids that encode vesicular stomatitis virus glycoprotein, NL-BH, and shRNAs. Two days after transfection, supernatants that contained viruses were collected and used to infect 293T cells in the presence of polybrene (8 μg/ml). After lentiviral infection of 293T cells, addition of puromycin (1 μg/ml) selected for cells that stably expressed shRNAs of KDM2B.

**FACS analysis**

Cells were trypsinized, washed, and fixed in 70% ice-cold ethanol overnight at 4°C. Immediately before flow cytometric analysis, the cells were treated with RNase (200 μg/ml) for 1 h at 37°C, stained with propidium iodide (PI, Sigma) for 30 min at RT, and subjected to fluorescence-activated cell sorting (FACS) analysis using a BD Accuri™ C6 Plus flow cytometer (BD Biosciences). First, sort gates were defined on an FSC vs. SSC dot plot to identify cells of interest based on cell size and to exclude most of the dead cells. Next, sort gates were defined on an FL2-A vs. FL2-H dot plot to count only the singlet population and to exclude aggregated cells. PI signal area (FL2-A) and height (FL2-H) parameters were applied to analyze the cell cycle of PI-stained cells. As the main parameter, histogram plot of FL2-A was used for a cell cycle graph following X-axis alignment of a test group to a control group. Data were analyzed using BD Accuri C6 software (BD Biosciences).

**Chromatin isolation**

Cells were lyzed by suspending in buffer A (10 mM HEPES, pH 7.9, 10 mM KCl, 1.5 mM MgCl_2_, 0.34 M sucrose, 10 % glycerol, 1 mM DTT, 0.1 % Triton X-100, protease inhibitor cocktail) and incubating on ice for 8 min. Nuclei were isolated by centrifugation at 4℃ for 5 min at 1,300 g and the supernatant was discarded. The resulting nuclei pellet was washed once with buffer A without TritonX-100, resuspended in buffer B (3 mM EDTA, 0.2 mM EGTA, 1 mM DTT, protease inhibitor cocktail), and incubated for 30 min on ice. The suspension was centrifuged at 4℃ for 5 min at 1,700 g and then the pellet was washed once with buffer B. The chromatin pellet was sonicated in buffer B and subjected to analysis of chromatin-associated proteins.

**Isothermal titration calorimetry (ITC)**

Isothermal titration calorimetry (ITC) experiments were carried out using an Auto-iTC200 Microcalorimeter at Korea Basic Science Institute. Tri-methyl H3K79 peptides, AQDFK(me3)TDLR, and unmodified H3K79 peptides, AQDFKTDLR, were purchased from AnyGen. Protein GST-PCNA was purified and prepared in the sample cell, and the ligand (H3K79 peptide) was loaded into the injectable syringe. All samples were prepared in 1X PBS. Titration measurements that consisted of 19 injections (2 μL) with 150 s spacing were performed at 25°C, while the syringe was stirred at 700 rpm. The data were analyzed using the MicroCal Origin^TM^ software.

**Chromatin immunoprecipitation (ChIP)-**

Formaldehyde (1%) was added to the medium for 10 min at room temperature, followed by the addition of 125 mM glycine for 5 min at room temperature. Adherent cells were scraped from dishes into 1 mL PBS. The scraped cells were centrifuged, and the resulting pellets were washed once with PBS. The pellets were resuspended in sodium dodecyl sulfate (SDS) lysis buffer (1% SDS, 10 mM EDTA, and 50 mM Tris-HCl, pH 8.1). The cell lysates were sonicated, diluted with five volumes of dilution buffer (0.01% SDS, 1.2 mM EDTA, 1.1% Triton X-100, 167 mM NaCl, and 16.7 mM Tris-HCl, pH 8.1), and incubated overnight with indicated antibodies (1 μg antibody for each IP reaction). The next day, protein A/G-Agarose beads (GenDEPOT) were added to the reaction, incubated for 2 h, and washed with low salt wash buffer (0.1% SDS, 2 mM EDTA, 1% Triton X-100, 150 mM NaCl, and 20 mM Tris-HCl, pH 8.1), high salt wash buffer (0.1% SDS, 2 mM EDTA, 1% Triton X-100, 500 mM NaCl, and 20 mM Tris-HCl, pH 8.1), LiCl immune wash buffer (1% SDC, 1 mM EDTA, 1% NP-40, 0.25 M LiCl, and 10 mM Tris-HCl, pH 8.1), and Tris-EDTA (TE) buffer (1 mM EDTA and 10 mM Tris-HCl, pH 8.0). The immunoprecipitates were eluted with elution buffer (1% SDS and 100 mM NaHCO_3_), incubated for 15 min twice, and reverse cross-linked at 65°C. Afterwards, the DNA fragments were purified for polymerase chain reaction (PCR) amplification. To analyze ChIP results, each enrichment level was normalized to input DNA. The means ± SD of the three independent experiments were represented as values.

**qPCR analyses**

The immunoprecipitated DNA fragments were purified and PCR-amplified for quantification, using each PCR primer pair. Disassociation curves were generated after each PCR run to ensure amplification of a single product. The mean threshold cycle (C_T_) and standard error values were calculated from individual C_T_ values, obtained from duplicate reactions per stage. The normalized mean C_T_ value was estimated as ΔC_T_ by subtracting the mean C_T_ of the input. To analyze replication origin loci of *JUNB*, *TOP1*, *CTCF*, *HBB*, Intergenic origin 1, and Intergenic origin 2 primer sets indicated in Table S1 were used. The primer concentration used for qPCR (Bio-Rad) was 0.2 μM*/*10 μL. The thermal cycler conditions were as follows: 15 min of holding at 95°C, followed by 39 cycles at 94°C for 15 s, 56°C for 30 s, and 72°C for 30 s.

**MTT assay**

shCTL and shKDM2B stable cells were seeded in 48-well plates (5 × 10^3^ cells/well). After 24, 48, 72, and 96 h, MTT was added to the cells (200 µL, final concentration 0.5 mg/mL), and the cells were incubated for a further 1 h at 37 ℃. The medium was then removed by aspiration, and DMSO was added (200 µL). The absorbance at 590 nm was determined using a spectrophotometer.

**Flow cytometric analysis of cell proliferation**

Flow cytometry analysis to measure cell proliferation were performed as described previously (Shi *et al*, 2015) with some modification. pLenti-GFP or pLenti-GFP-KDM2B plasmids were used to generate lentivirus by transfecting 293T cells with pMD2.G and pPAX2 plasmids using PEI reagent. Viral supernatants were collected between 36 and 72 hour time points following transfection. For proliferation assays, H3-WT and H3-K79R stable cells were subjected to GFP lentiviral transduction and flow cytometry analysis using a BD Accuri™ C6 Plus flow cytometer (BD Biosciences). Gating was performed on live cells using forward and side scatter, prior to measuring of GFP positivity.
